# Investigation of the efficacy of two different laser types in the treatment of lower lip paresthesia after sagittal split ramus osteotomy

**DOI:** 10.1007/s10103-024-03973-9

**Published:** 2024-01-09

**Authors:** Ebru Baydan, Emrah Soylu

**Affiliations:** Department of Oral and Maxillofacial Surgery, Erciyes University Faculty of Dentistry, Kayseri, Turkey

**Keywords:** Sagittal split ramus osteotomy, Inferior alveolar nerve damage, Paresthesia, Low-level laser therapy

## Abstract

Orthognathic surgery involves invasive and major surgical procedures commonly used to correct maxillofacial deformities. Bilateral sagittal split ramus osteotomy (BSSO) is often used to treat dentofacial anomalies related to the mandible, but it can result in various complications, the most common of which is inferior alveolar nerve damage. Nerve damage–induced paresthesia of the lower lip significantly affects patient comfort. Medical treatments such as steroids and vitamin B, low-level laser therapy (LLLT), and platelet-rich fibrin (PRF) can be used as supportive therapies for nerve regeneration after damage. This study aimed to investigate the effectiveness of two different types of lasers in treating lower lip paresthesia after BSSO. This clinical trial was a controlled, single-center, prospective, single-blind, randomized study. Thirty patients were included in the study and randomly assigned to three groups: Group I (laser GRR, *n* = 10) received transcutaneous and transmucosal GRR laser treatment, Group II (Epic10 laser, *n* = 10) received transmucosal and transcutaneous Epic10 laser treatment, and Group III (vitamin B, *n* = 10) received B-complex vitamin tablets orally once a day. Two-point and brush tests were performed six times at specific intervals, and a visual analog scale was used to evaluate pain and sensitivity. Both vitamin B and laser therapies accelerated nerve regeneration. The contribution of the laser groups to the healing rate was better than that of the vitamin B group. Although there was no statistically significant difference between the two laser groups, clinical observations indicated better results in the GRR laser group.

## Introduction

Orthognathic surgery is performed in cooperation with orthodontics and maxillofacial surgery to correct dentofacial deformities and to establish the correct skeletal relationship in patients who have completed growth development. Orthognathic surgery aims to restore lost function, phonation, and esthetics in patients. An important component of orthognathic surgery is bilateral sagittal split ramus osteotomy (BSSO), which is often performed with upper jaw surgery or alone.

BSSO was introduced by Trauner and Obwegeser [1] in 1957 and is currently frequently used for the treatment of dentofacial anomalies associated with the mandible. Shortly after the introduction of BSSO, several important and widely used modifications were proposed by Dal Pont (1961), Hunsuck (1968), and Epker (1977). Although many modifications have been identified from the past to the present to reduce the complications caused by this surgery, many unwanted complications may still occur after surgery.

Studies have revealed that various complications may develop related to the orthognathic surgical procedure, with nerve damage being the most common [2]. Paresthesia of the lower lip due to the inferior alveolar nerve (IAN) damage in the postoperative period after BSSO can cause an inability to talk, saliva leakage, or lip chewing and significantly affects patient comfort [3]. Therefore, management of the paresthesia process that is likely to occur is important. Medical (steroid, vitamin B), low-level laser (LLL), and platelet-rich fibrin (PRF) treatments can be applied as supports in the regeneration process after nerve damage. LLL has been used in many fields of medicine and dentistry. It has been proven by studies that it accelerates tissue healing by acting through different mechanisms [4]. LLL increases blood flow in the applied area; collagen synthesis provides an increase in cell respiration, and adenosine triphosphate (ATP) synthesis provides an anti-inflammatory effect and causes an increase in venous and lymphatic flow, and as a result, swelling disappears quickly. It also provides an analgesic effect, supports the regeneration of nerve cells, accelerates wound healing, increases myelin production capacity, and accelerates axonal growth [5–8].

The vitamin B complex is also one of the treatments that accelerate nerve regeneration. The vitamin B complex contains B1, B2, B6, B9 (folic acid), and B12. B-group vitamins are an important micronutrient required in many biological processes [9].

Thiamine (B1), pyridoxine (B6), and cobalamin (B12), which are also known as neurotropic vitamins, maintain neuronal vitality. In healthy nerve cells, vitamin B1 acts as an antioxidant, while vitamin B6 balances nerve metabolism, and vitamin B12 protects myelin sheaths. In case of nerve damage, the presence of vitamins B1, B6, and B12 supports the development of new cell structures and opens the way for regeneration. Additionally, vitamin B1 facilitates the use of carbohydrates for energy production, whereas vitamin B12 supports the survival and remyelination of nerve cells. The absence of these vitamins will lead to permanent nerve degeneration and ultimately peripheral neuropathy [10].

Due to the synergistic effect of vitamins B1, B6, and B12 on nerve cell damage, it has been shown in studies that supplementation of these vitamins in the acute period may be useful for accelerating nerve regeneration [11].

This study aimed to investigate the efficacy of two different laser types in the treatment of paresthesia of the lower lip after BSSO and to compare this efficacy with that of the vitamin B complex.

## Material and methods

### Study design

This study was a prospective, randomized, controlled clinical trial. The study was approved by the Ethics Committee of Erciyes University (23/09/2020 – 2020/479) and the Ministry of Health of Turkiye Republic (18/03/2021 – 2020/127) and was conducted at the Department of Oral and Maxillofacial Surgery of the Erciyes University Faculty of Dentistry. All volunteers were informed about the laser devices to be used in the study and the drug application, possible side effects, and complications, and informed consent was obtained.

### Sample size calculation

Using the data reported in the study of Sharifi et al. [17], it was determined that there should be at least 10 individuals in each group, according to the power analysis performed with the G*Power (ver. 3.1.9.7, Heinrich Heine Universitat Düsseldorf, Germany) software according to *d* = 1.375 (large), alpha = 0.05, and 90% power parameters [12].

### Selection criteria

Thirty patients who underwent BSSO and experienced sensory changes in the lower lip due to nerve damage 3 weeks after surgery were included in the study. Patients with systemic disorders affecting nerve healing, neurological and psychiatric medications, patients who had previous surgery or trauma in the mandibular region, preoperative neurosensory dysfunction in the IAN, and simultaneous genioplasty surgery were excluded from the study. The patients were randomly divided into three groups as follows: GRR laser (*n* = 10), Epic10 laser (*n* = 10), and vitamin group (*n* = 10).

Cone-beam computed tomography (CBCT) scans of the patients were examined before the surgery. The course of the IAN was noted. Patients were informed about all possible complications of the operation, including the risk of nerve damage. Informed consent forms were signed by all patients. Clinical examinations and laser applications of the patients were performed by the same researcher.

### Surgical procedure

All patients were anesthetized by the same anesthesia team in the general operating room of Erciyes University Oral and Maxillofacial Surgery Hospital and operated by the same surgical team. Following nasotracheal intubation, bilateral buccal, inferior alveolar, and lingual nerve blocks were performed in the mandible with 2% articaine 80 mg + 1/200,000 epinephrine (Ultracaine 2%, Ampule, Sanofi Aventis, Istanbul, Turkey) for all patients. BSSO was performed according to the Hunsuck’s [13] modification and the mandible was placed into its pre-planned position, and mini-plates and titanium mono-cortical screws were used for fixation.

If nerve damage occurred during surgery, the type of nerve damage was recorded according to the Seddon classification [14].

### Evaluation of neurosensory disturbances

The impact of surgery-induced edema on immediate postoperative sensory changes in patients was significant. Therefore, a standard steroid treatment (dexamethasone) was administered to the patients to mitigate the effects of edema on the inferior alveolar nerve sensory function. The treatment involved taking 4 mg of dexamethasone three times on the surgery day, followed by 4 mg twice a day on the second day, and 4 mg once a day on the third day. Patients were monitored for 3 weeks after the operation, and 30 patients who reported lower lip paresthesia/numbness were included in the study. Neurosensory tests were conducted on each patient before commencing the prescribed treatment.

### Neurosensory test protocol

Direction determination, two-point separation, and pinprick tests were performed on the patients, along with the brush test following the protocols described in the literature [13]. These neuro-sensitivity tests were conducted in the study group before treatment (T0), on the third day of treatment (T1), on the fifth day of treatment (T2), on the seventh day of treatment (T3), on the ninth day of treatment (T4), and after the end of treatment (T5). In the control group, these tests were conducted before treatment (T0), in the first week of treatment (T1), in the second week of treatment (T2), in the third week of treatment (T3), in the fourth week of treatment (T4), and after the end of treatment (T5). Each patient underwent six neurodevelopmental tests. A neurodevelopmental test was conducted in the laser groups before laser application.

**Fig. 1:**
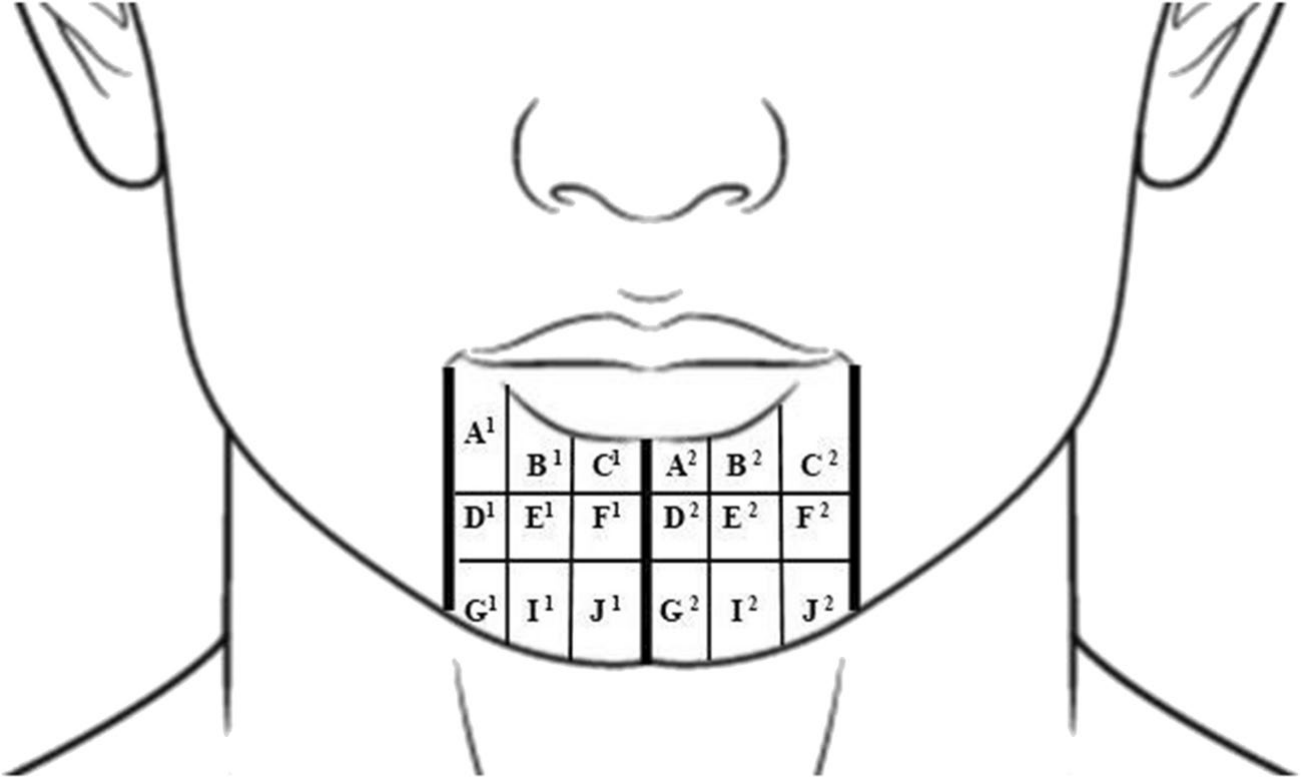
The area between the lower lip and the tip of the chin was divided into 9 zones

Before the neurosensory test was started, the test to be performed was explained in detail. The patient’s eyes were closed during the test. After identifying the area of the patient with loss of sensation (right and left), the area between the lower lip and the tip of the jaw was divided into nine areas for each side (Fig. 1). The patient’s two-point separation, ability to determine the direction with the brush test, and perception of pain and pressure were examined in these areas. The data obtained from these tests were recorded on patient-specific forms.

First, the examination began with a brush test and direction determination. This was recorded as “yes” or “no”. The examination was continued by using a two-point separation test. The minimum interval in which the patient could distinguish between the two points was recorded in millimeters. Finally, the examination was terminated by using the pinprick test, which measures the sensation of pain. The tip of the probe was inserted into each area on the side where the patient described paresthesia (right, left, or both sides). The patient was asked to indicate the severity of pain sensation based on the Visual Analog Scale (VAS) score, and these values were recorded. The sections on the VAS used to determine the severity of pain and pressure were as follows: 1 (complete absence of sensation), 2 (almost no sensation), 3 (decreased sensation), 4 (almost normal sensation), and 5 (completely normal sensation).

### Treatment protocol

#### Group I (GRR laser group)

**Fig. 2:**
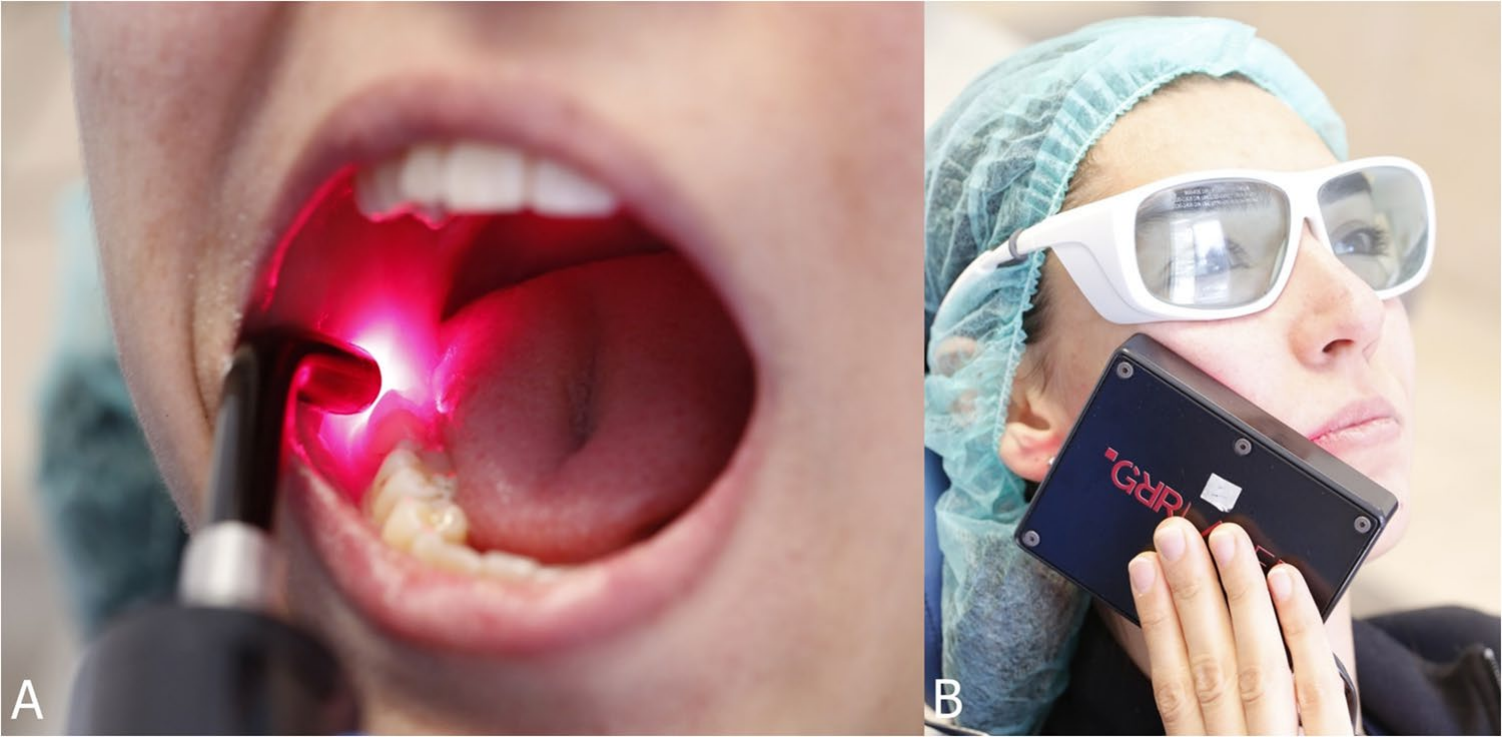
Intraoral (**A**) and extraoral (**B**) clinical applications of GRR laser

GRR laser (GRR Laser Medical Ltd. Şti, Ankara, Turkey) is a gallium aluminum arsenide (GaAlAs) laser combined with light-emitting diode (LED) that utilizes a combination of wavelengths of 904 and 650 nm, with a penetration depth of 50 mm and includes two probes for oral and extra-oral applications, with the intraoral probe featuring a 10-mm diameter and an energy transfer of 9 J during a 1-min application. The external probe had an external area of 60 mm and an internal area of 30 mm, with an energy transfer of 16 J during a 1-min application. In Group I, 10 patients received 10 sessions of GRR laser treatment over 5 weeks, with transmucosal and transcutaneous lasers applied twice a week. Each session lasted 10 min. Protective glasses were worn by the patients before laser application. A transmucosal laser was then applied for 5 min, starting from the lingula region in the mouth and along the incision line to the point where the mental nerve exited. Outside the mouth, a rectangular probe was placed between the ramus and jaw tip, and a 5-min transcutaneous laser was applied. The procedure was performed according to the recommendation of the manufacturer (Fig. 2).

#### Group II (Epic10 laser group)

**Fig. 3:**
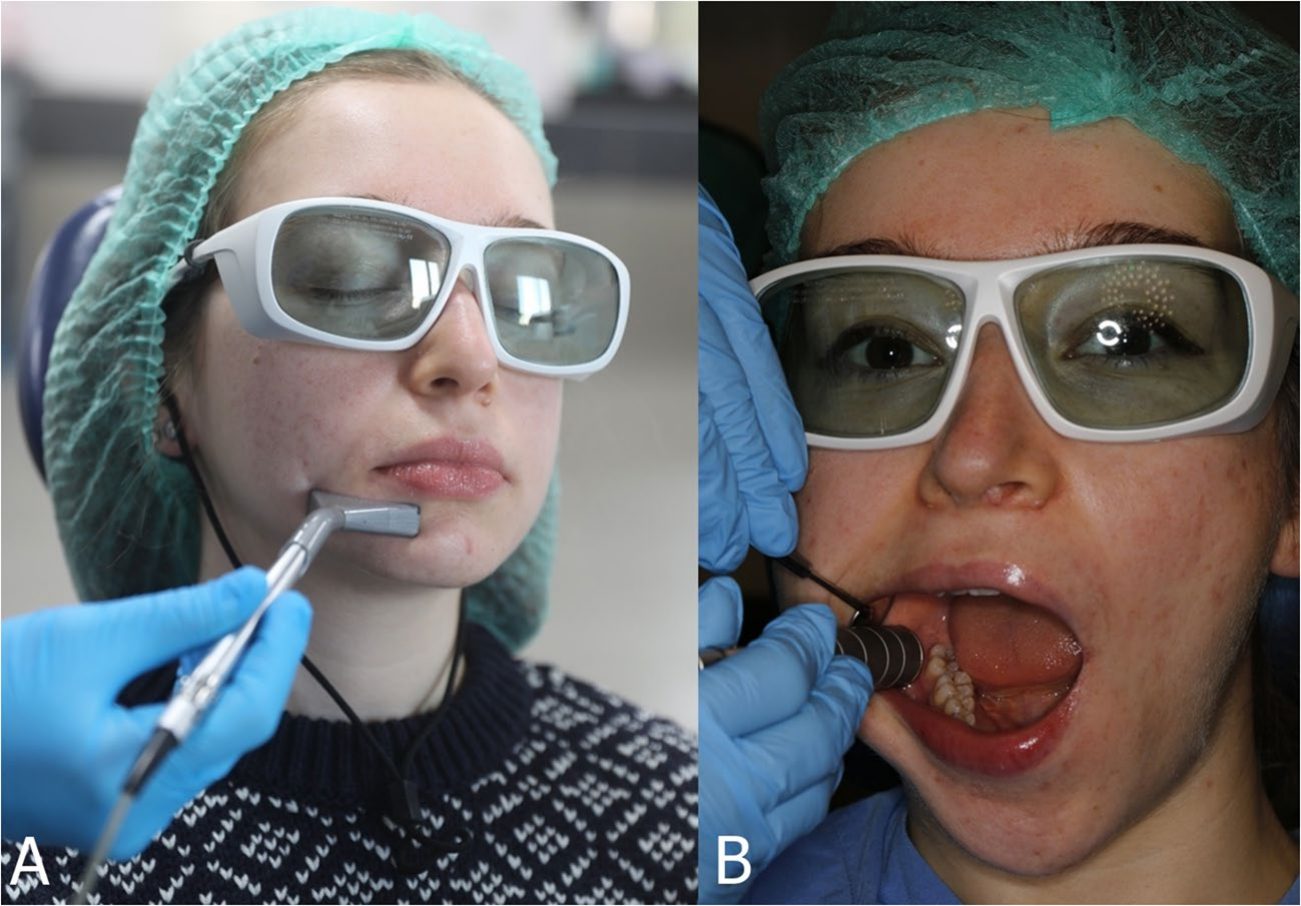
Intraoral (**A**) and extraoral (**B**) clinical application of Epic10 laser

Epic10 laser (Biolase Inc. California, USA) is a GaAlAs laser that operates at a wavelength of 940 nm and an energy density of 5 J. The area of the external probe was 2.8 cm^2^ (35 mm × 8 mm), whereas the intraoral probe had a diameter of 15 mm. The laser penetration depth was 5 mm. All 10 patients in this group underwent 10 sessions of Epic10 laser treatment twice a week, using a transmucosal and transcutaneous application, targeting the side with neurosensory disturbances (NSD). The probe was continuously moved during the application to prevent any damage to the soft tissue owing to the heat generated during the procedure. The treatment lasted for 5 weeks, with each session consisting of a 10-min laser application to the area with NSD. Protective glasses were worn by the patients during laser application to ensure ocular safety. A transmucosal laser was applied for 5 min, starting from the lingula region in the mouth and following the incision line up to the exit point of the mental nerve. Additionally, a transcutaneous laser was applied extraorally for 5 min. The procedure was performed according to the recommendation of the manufacturer (Fig. 3).

#### Selection criteria for laser devices

GRR laser and Epic10 laser devices are in the inventory list of the department. GRR laser is a recently developed device by an oral and maxillofacial surgeon and was approved by the Ministry of Health of the Turkiye Republic. The manufacturer claims that this device can be used to treat temporomandibular joint pain, myalgia, medical-related osteonecrosis of the jaws (MRONJ), trigeminal neuralgia, facial paralysis, trismus, oral aphtha and ulcers, alveolitis, back pain, postoperative paresthesia of the lower lip, and bruxism. The Epic10 laser has been used in the department for a decade for the treatment of lower lip paresthesia related to wisdom tooth removal or BSSO and for frenectomy. It aimed to investigate the claimed effects of GRR laser on post-operative paresthesia compared with the Epic 10 laser. The differences between the two laser devices are listed in Table 1.

**Table 1:** Differences between two laser devices

	GRR laser	Epic10 laser
Wavelength	904–650 nm	940 nm
Prob diameter	10 mm	15 mm
Energy density	9 J	5 J
Penetration depth	50 mm	5 mm
Application	Stable probe	Moving probe

#### Group III (vitamin group)

As it was deemed unethical to administer a placebo treatment to the control group, vitamin therapy was administered to the patients in this group. Therefore, in addition to comparing the effectiveness of the two different lasers, the recovery rates of the vitamin B group and the laser-applied groups were also compared. The patients in this group received oral administration of vitamin B complex (Bemiks 30 tablets, Zentiva Health Products Co. LTD, Kırklareli, Turkiye) which contained 10 mg of thiamine-B1, 2 mg of riboflavin-B2, 2 mg of pyridoxine-B6, 20 mg of niacinamide, 3 µg of vitamin B12, 2.5 mg of folic acid, and 5 mg of calcium pantothenate, once a day for 30 days.

### Statistical analysis

The suitability of the data for normal distribution was evaluated by histogram, q–q graphs, and Shapiro–Wilk test. The variance homogeneity was tested with the Levene test. In the comparisons between more than two groups, Kruskall–Wallis variance analysis was used for quantitative variables. Pearson *χ*2 analysis was used for the comparisons of categorical data. The Dunn test was used for multiple comparisons. Data analyses were performed with an online statistical software (TURCOSA, Turcosa Analytic Ltd Co., Turkiye, www.turcosa.com.tr). The significance level was accepted as* p* < 0.05.

## Results

The study included a total of 30 patients (22 female and 8 male) aged 18–40 (mean age of 23.43) who underwent BSSO surgery to correct skeletal deformity at Erciyes University Faculty of Dentistry, Department of Oral and Maxillofacial Surgery.

### Demographic findings

Three males and seven females were included in Group I, 10 females in Group II, and five females and five males in Group III. No significant relationship between gender and groups was found (*p* = 0.053). The mean age of patients in Group I was 20 years, 27 years in Group II, and 19.5 in Group III. The Kruskal–Wallis test indicated a significant difference in age distribution among the groups (*p* = 0.007).

The IAN damage during surgery was classified according to the Seddon classification as follows: “Type 0” if there was no damage to the nerve, “Type 1” if neuropraxia developed, and “Type 2” if axonotmesis developed. Patients who developed neurotmesis were excluded from the study.

The nerve damage type distribution of patients in Group I was as follows: “Type 0” for 0 patients, “Type 1” for 5 patients, and “Type 2” for 5 patients. In Group II, 4 patients had “Type 0”, 3 patients had “Type 1”, and 3 patients had “Type 2” nerve damage. In Group III, 3 patients had “Type 0”, 5 patients had “Type 1”, and 2 patients had “Type 2” nerve damage.

According to the statistical analysis, there was no significant difference between the types of nerve damage and the groups (*p* = 0.204).

### Clinical examination findings

#### Brush test (BT)

If a patient responded positively to the brush test during the direction determination test, it was recorded as “yes (1)”, while a negative response was recorded as “none (0)”. Most patients were able to determine the direction using the brush test, and those who were initially unable to do so were able to after the second examination. After analyzing all six examinations, no significant difference was found between the results of the BT examination and the groups (*p* > 0.05).

#### Two-point discrimination test (TPD)

In the first three examinations, there was no significant difference between the group categories in terms of the amount of two-point discrimination of the patients (*p* > 0.05). After the 4th examination, there was a significant difference between the laser groups and the vitamin group in terms of the amount of two-point discrimination among the patients (*p* = 0.028). (Table 2).

**Table 2:** Relationship between groups of T3 test results

Groups	*N*	Middle	25%	75%	Lowest	Biggest	Mean rank	
GRR laser (group I)	10	2	2	2	1	25	13.5	***P* = 0.028**
Epic10 laser (group II)	10	2	2	2	2	2	13	
Vitamin group (group III)	10	2.5	2	3.5	2	15	20	

Values were given in mm

#### Pinprick test

##### Comparison between groups

The patient registration form included sections on the VAS scale to determine the severity of pain and pressure. The scale ranged from 1 (complete absence of sensation) to 5 (completely normal sensation). The lower lip was divided into nine regions, and each of these regions was labeled. (see Fig. 1) The patient was asked to indicate the severity of the pain sensation they felt when given a painful stimulus to each area, considering the VAS score, and these values were noted. The results of six separate examinations of each point were compared between the groups.

There was a significant difference between the GRR laser–vitamin groups in the sixth examination results of the pain sensation test of the C point (*p* = 0.039). Significant differences were also found in the 6th examination of the C point (*p* = 0.029) (Table 3). However, no significant difference was found in the examination comparisons of the other points (*p* > 0.05).

**Table 3:** The relationship between the groups and the T7 VAS scores of the pain sensation test of the C point

Groups	*N*	Middle	25%	75%	Lowest	Biggest	Mean rank	
GRR laser (group I)	10	5	3.75	5	3	5	19.1	***P* = 0.029**
Epic10 laser (group II)	10	5	2.75	5	1	5	17.6	
Vitamin group (group III)	10	4	1.75	4	1	4	9.8	

##### Intra-group comparison

The examination findings at T0 and at T5 were compared within each group, separately for each point. Therefore, the effectiveness of the treatments in each region was compared.

##### Point A

All three treatment groups showed significant differences in the examination findings of the “A” point at T0 and T5 (*p* < 0.05).

##### Point B

There were significant differences between the examination findings of the “B” point at T0 and T5 in all 3 treatment groups (*p* < 0.05).

##### Point C

While there were significant differences between the examination findings of the “C” point at T0 and T5 in the laser groups (*p* < 0.05), there was no significant difference in the vitamin group (*p* = 0.090).

##### Point D

There were significant differences in the examination findings of the “D” point at T0 and T5 in the laser groups (*p* < 0.05). However, no significant difference was observed in the vitamin group (*p* = 0.121).

##### Point E

There were significant differences between the examination findings of the “E” point at T0 and T5 in all 3 treatment groups (*p* < 0.05).

##### Point F

While there were significant differences between the examination findings of the “F” point at T0 and T5 in the GRR laser group (*p* < 0.001), there was no significant difference in the Epic10 laser and vitamin groups (*p* = 0.090).

##### Point G

There were significant differences between the examination findings of the “G” point at T0 and T5 in all 3 treatment groups (*p* < 0.05).

##### Point I

There were significant differences between the examination findings of the “I” point at T0 and T5 in all 3 treatment groups (*p* < 0.05).

##### Point J

There were significant differences between the examination findings of the “J” point at T0 and T5 in all 3 treatment groups (*p* < 0.05).

## Discussion

Orthognathic surgery is a major surgical procedure commonly used to correct dentofacial deformities by making invasive changes [16]. The most common surgical procedure for the mandible is BSSO. Despite numerous modifications made to prevent complications associated with BSSO, many risks still remain. One of the most common complications is injury to the IAN and the severity of the damage can range from neuropraxia to neurotmesis, with the latter requiring microsurgical repair [2, 15, 17]. In the literature, the incidence of IAN injury after BSSO ranges from 9 to 85% [18, 19]. The neurosensory changes (NSC) after BSSO are usually a combination of axonotmesis and neuropraxia [20], with the lower lip and chin tip being the most affected areas [21]. These sensory changes can cause significant functional impairments in speech and eating, leading to social and psychological consequences. In a study by Sandstedt et al. [22], more than 70% of patients with nerve damage complained of paresthesia, and at least one in five patients experienced pain in the affected area. In the present study, all included patients complained of lower lip paresthesia without neurologic pain 3 weeks after the surgery, and laser probes were applied to the areas innervated by the IAN as the retromolar region corresponding to the mandibular foramen, mental foramen, lower lip, and skin of the anterior mandible. This procedure was performed in 10 sessions, twice a week, as recommended in the literature [1, 23, 24].

Although cone-beam computed tomography (CBCT) of all patients was taken and all images were examined prior to surgery, patient-specific factors such as the anatomy of the mandible, course of the IAN in the bone, surgeon’s experience, surgical method, amount of activation, type of fixation, and presence of a wisdom tooth in the surgical field can increase the risk of complications [2, 25–28]. It is recommended that impacted wisdom teeth should be removed at least 6 months before the surgery to reduce the risk of complications related to IAN injury [26]. The patients included in the present study had their third molars removed at least 6 months before BSSO. To standardize the fixation type, osteotomized fragments were fixed with mini-plates and mono-cortical screws in all patients included in the present study.

The incidence of NSC after BSSO has led to studies identifying risk factors [27, 28]. In a study by Demirbas et al., patients were categorized based on age, gender, type of deformity, nerve manipulation, right and left mandibular translation, and amount of mandibular movement, and the correlation between these factors and NSC recovery time was investigated. They found that NSC recovery time was significantly associated with patient age, amount of mandibular movement (> 7 mm), and nerve manipulation, while no significant correlation was found with gender, type of deformity, and the right and left mandibular movements [29]. The present study included patients who developed NSC 3 weeks after BSSO and aimed to compare the effectiveness of three different treatment options on NSC. The focus of the present study was to assess the efficacy of the treatments in curing NSC and factors that may have contributed to the development of NSC, such as the type of deformity, nerve manipulation during surgery, or the movement of the left and right sides of the mandible, were not documented.

Various treatments have been developed to accelerate nerve regeneration and shorten the NSC recovery time. The use of GaAsAl laser for the treatment of neurosensory changes after BSSO has been shown to be effective in restoring normal function [29–33]. The degree of neurosensory damage can affect the success of treatment. In a study by Guarini et al. [21], all patients who received laser therapy reported better neurosensory recovery than those who did not receive laser treatment. Numerous studies have established the effectiveness of laser therapy for nerve regeneration [4, 13, 15, 17, 34]. The aim of the present study was to compare a recently developed laser device with two different wavelengths with a single-wavelength laser device that is proven to be effective in lower lip paresthesia treatment.

The penetration ability of lasers can vary based on their wavelengths, and the double wavelength (904/650 nm) of the GRR laser used in the present study increased the penetration ability by approximately 10 times. One of the objectives of the present study was to investigate whether increasing penetration ability enhances the effect of nerve regeneration. The results of the present study showed a statistically significant difference between the results of examinations conducted before and after laser therapy in patients with improved neurosensory function. Although there was no statistical difference in the contributions of the two lasers to recovery when compared with each other, some patients in the GRR laser group reported a VAS score value of “5” during examinations at the end of treatment, whereas some patients in the Epic10 laser group reported a VAS score value of “4”. This relative superiority of the GRR laser to the Epic10 laser can be attributed to the higher penetration depth of the GRR laser. Additionally, the Epic10 laser probe should be used with continuous movement to prevent an increase in the skin and tissue temperature, which can result in a reduction in the planned dose transferred to the related tissue or soft tissue damage.

In the present study, each side of the lower lip and chin area was divided into nine regions (Fig. 1), the NSC was not the same for each sub-region, and recovery did not occur at the same rate. The results of the present study showed that the area with the most neurosensory disorders was observed at the “C” point, situated in the middle and upper parts of the lower lip. The comparison of the results between groups showed that the GRR laser group responded best to the treatment applied at point “C”. It was thought that the penetration depth of the GRR laser is responsible for the recovery of the “C” point, as the most distal part of the IAN.

Although vitamins B1, B6, and B12 individually exhibit nerve-regenerating effects, studies have shown that combining them provides synergy and, thus, supports nerve regeneration more effectively [35]. Jolivalt et. al. [36] conducted a study that demonstrated the combination of vitamins B1, B6, and B12 to be more effective for sensory nerve function in experimental diabetic rats than individual B vitamins in a dose-dependent manner. In another study conducted by Fujii et al. [37], high doses of a combination of B1, B6, and B12 resulted in stronger in vitro nerve growth of murine dorsal root ganglia compared to combinations in which only one of the three vitamins was a high dose. In the present study, due to ethical concerns of the authors, the control group took 1 tablet of vitamin B complex each day for 30 days, instead of a placebo.

Another aim of the present study was to compare the effects of laser and vitamin B therapy on damaged nerves. The results of this study showed that there were statistically significant differences in the A, B, E, G, I, and J regions (Fig. 1) when the pre- and post-treatment examinations of patients receiving vitamin B complex as the control group were compared. These findings support the existing literature and confirm the regenerative effects of vitamin B on nerve damage. Comparing the laser groups with the control group, the statistical analysis showed that laser treatment increased and supported nerve regeneration more effectively. As a result of the study, laser therapy was initiated for patients in the control group who reported paresthesia that did not exceed NSC. In postoperative examinations of the 30 patients included in the study, it was found that their paresthesia had completely resolved 6 months after the surgery.

The treatment protocol of the laser devices was discussed in the literature [1, 23, 24]. In the present study, the laser treatment was applied to the patients in the laser groups two times a week for 5 weeks as a total of 10 appointments. This protocol was applied by following the recommendations of the literature and the manufacturers of the devices. Although laser treatments have proven to be effective in paresthesia recovery, this treatment protocol is not suitable for patients coming from another city, and it is challenging for patients to attend treatment appointments. Thus, vitamin B treatment can be a more useful approach for these kinds of patients.

The healing of nerve damage can be assessed using subjective or objective examinations. Agbaje et al. [38] concluded in their study that subjective evaluations are the most common approach for evaluating neurosensory deficits. In the present study, consistent with other studies in the literature, subjective tests were used to evaluate neurosensory function in patients. These included the brush, two-point discrimination, and pinprick tests that were evaluated using VAS scores. The treatment of 30 patients with NSC was initiated 3 weeks after the operation. The electrical susceptibility testing of mandibular molars was not used in the present study since it is a reliable evaluation only up to the 4th postoperative day [39]. Furthermore, the thermal test was not used since it was reported in the literature that it is not specific enough to distinguish between normal and abnormal thermal sensations, and laser treatments did not have a significant effect on the neurosensory recovery of thermal sensation [17, 40].

The present study was performed on a relatively low number of patients. Also, as the authors aimed to investigate the effect of laser therapy on the recovery of IAN damage, the classification of skeletal relation (Cl 2 or Cl 3), the amount of mandibular movement, the direction of mandibular movement (set-back or advancement), and nerve manipulation during surgery were not included. These factors are considered the limitations of the present study.

## Conclusion

In conclusion, the findings of the present study supported the existing literature that both vitamin B and laser therapy can promote nerve regeneration. However, the results of the present study showed that laser therapy was more effective than vitamin B therapy in promoting nerve regeneration. Although there was no significant difference between the two laser groups, patients treated with GRR laser showed better clinical outcomes. Increasing the sample size is recommended in future studies to provide a better understanding of the differences between these two laser devices. This study is the first of its kind to examine and compare the effectiveness of two different laser devices with distinct wavelengths on nerve regeneration and to compare this effect with vitamin B therapy.
